# Influence of a multidimensional music-based exercise program on selected cognitive and motor skills in dementia patients—a pilot study

**DOI:** 10.1007/s12662-021-00765-z

**Published:** 2021-10-15

**Authors:** A. Prinz, A. Schumacher, K. Witte

**Affiliations:** Department of Sport Science, Otto-von-Guericke University Magdeburg, Zschokkestr. 32, 39104 Magdeburg, Germany

**Keywords:** Dementia, Cognition, Motor performance, Music-based exercise program

## Abstract

One of the most common causes of needing care in old age is dementia. In order to enjoy a pleasant retirement for people with dementia, it is essential for them to maintain their independence. Studies have shown that a combination of physical activity and music has positive effects on dementia patients. Therefore, this study aimed to investigate the feasibility of implementing a multidimensional music-based exercise program for dementia patients and the effects on an intervention group (IG) compared to a control group (CG, usual care). The study design was based on a 12-week intervention with two (IG/CG) by two (pretest/posttest) parallel groups and block randomization with unequal group sizes. Participants had to be able to move independently or with a walker and not have severe cardiovascular disease or cardiac arrhythmias. Fifty-three blinded dementia patients (age: 83.63 ± 6.03 years) from inpatient facilities participated in the study and were assigned from the exercise instructors to IG (*n* = 34) and CG (*n* = 19). The primary outcomes were feasibility (Observation sheet), modified Chair Rising test, Timed Up and Go test, hand dynamometer test, FICSIT‑4 (Frailty and Injuries: Cooperative Studies of Intervention Techniques), and drop bar test. Secondary endpoints included: verbal fluency (“animals”), the Mini-Mental State Examination, memory, Trail Making Test A, and Qualidem. Forty-nine subjects were analyzed (IG = 32; CG = 17). There were significant differences between the groups in the modified Chair Rising test (*p* = 0.033), FICSIT‑4 (*p* = 0.035), and Timed Up and Go test (*p* = 0.005) at posttest, which showed improved performance of the IG compared to the CG. The IG additionally showed improvements in the modified Chair Rising test (*p* = 0.000), drop bar test (*p* = 0.033), hand dynamometer test (*p* = 0.001), Timed Up and Go test (*p* = 0.000), verbal fluency (*p* = 0.002) and Trail Making Test A (*p* = 0.04) after 12 weeks. There were no adverse events or side effects. The multidimensional music-based exercise program could be performed by the dementia patients and was well received. The improved functional mobility could contribute to a lower risk of falls and thus maintain independence. For the follow-up study, the number of subjects, randomization, and parameters should be considered.

**Supplementary Information:**

The online version of this article (10.1007/s12662-021-00765-z) contains supplementary material, which is available to authorized users.

## Introduction

Dementia is a neurodegenerative disease that is associated with a decline in cognitive and motor performance. It proceeds in a gradual process, and in most cases, leads to a loss of independence and the need for care (DGPPN, 2017). Thus, dementia is also a social problem, as demographic change and the resulting increase in dementia illnesses imply high costs for society and health insurance companies (Michalowsky, Kaczynski, & Hoffmann, 2019). By 2050, Germany’s number of dementia patients will double from 1.6 million to almost 3 million (Deutsche Alzheimer Gesellschaft e. V. Selbsthilfe Demenz, 2014). As dementia results in a need for care, the number of people requiring inpatient care and the need for care in nursing facilities is also increasing (Robert Koch-Institut, 2015; Statistisches Bundesamt, 2018). The risk of falls is a major cause of this increase, and motor performance decrease due to dementia, resulting in loss of independence. There is already some evidence that various nonpharmacological forms positively affect people with dementia (Cabrera et al., 2015; de Oliveira et al., 2015). Despite these findings, dementia is preferentially treated with medication, regardless of the side effects (DGPPN, 2017). Due to the consequences of demographic change, however, it is essential to develop nonpharmacological interventions for dementia patients and to incorporate them into the daily lives of dementia patients to achieve effective dementia management (Kressig, 2017). The German Society of Psychiatry and Psychotherapy, Psychosomatics and Neurology also support this recommendation (DGPPN, 2017). Physical exercise interventions (Demurtas et al., 2020; Forbes, Forbes, Blake, Thiessen, & Forbes, 2015; Li, Guo, Wei, Jia, & Wei, 2019; Yeh et al., 2021) and music interventions (Leggieri et al., 2019; van der Steen et al., 2018) have been successful. Due to the limited mobility of seniors in nursing facilities and dementia patients, chair-based interventions are particularly recommended. These have also shown positive effects on motor and cognitive performance (Cordes, Schoene, Kemmler, & Wollesen, 2020; Cordes, Zwingmann, Rudisch, Voelcker-Rehage, & Wollesen, 2021). A review by Borges-Machado et al. (2020) also showed that a multidimensional design of training with light to moderate intensity, consisting of a combination of strengthening, coordination, balance, and flexibility, is preferable to focus-only training in dementia patients. Cordes et al. (2021) and Scharpf, Servay, and Woll (2013) demonstrated the positive effects of a combination of chair-based intervention with multidimensional content. Music interventions could also reach people with dementia in the same way. They positively influence the emotional, social, and personal levels (Brancatisano, Baird, & Thompson, 2019). Memories of various events are closely associated with music (Cloos, 2014). Music memory is the region in the brain preserved in dementia until the late stages and significantly impact dementia patients (Jacobsen et al., 2015). It has a motivating effect and can positively influence mood. Due to the positive results of exercise programs and music programs, studies examined the combination of both programs (Gomaa, Wittwer, Grenfell, Sawan, & Morris, 2018). The few studies that analyzed this combination provided evidence that it may be more effective on cognitive and motor performance in dementia patients than single music or a single exercise interventions (Marks & Landaira, 2015; Terry, Karageorghis, Curran, Martin, & Parsons-Smith, 2020). However, since the study situation is not uniform concerning individual procedures and the results of studies do not have sufficient evidence, further research is needed in this regard (Schilder & Philipp-Metzen, 2018). Especially the combination of music with exercise and the combination of music and strength endurance has been little researched so far (Gomaa et al., 2018). Therefore, it is essential to conduct further sound studies on nondrug treatments focusing on music-based exercise programs, as these could be essential for effective dementia management (Kressig, 2017). In addition, there is currently little guidance on what the basic training design for dementia patients should look like, for example, in terms of communication and motivation (Eggenberger, Heimerl, & Bennett, 2013; van Alphen, Hortobágyi, & van Heuvelen, 2016).

Therefore, this study aimed to investigate the feasibility and efficacy of a music-based exercise program with contents of a multidimensional chair-based intervention in dementia patients. The key domains investigated were motor function, cognition, and quality of life. This leads to the following two main research questions: (a) To what extent is this newly developed music- and chair-based exercise program feasible with dementia patients and is it accepted by them? (b) How does the program influence motor and cognitive performance and quality of life in people with dementia? According to the mentioned literature, we hypothesized that a music-based exercise program with contents of a multidimensional chair-based intervention is feasible in people with dementia and can improve and maintain motor performance (hypothesis 1) as well as cognitive performance and quality of life (hypothesis 2).

## Materials and methods

### Study design

The present study was designed as a 12-week pilot intervention study with two (group: intervention/control) by two (measurement time points: pre-/post-test) parallel-group designs with block randomization with unequal group sizes. The research protocol conformed to the principles of the Declaration of Helsinki and was approved by the Ethics Committee of Otto von Guericke University Magdeburg (Germany) (registration number: 100/20). Data collection took place in September 2020 (pretest) and February 2021 (posttest). The tests at the two-time points (pretest/posttest) were performed at the respective facilities. The intervention was conducted in the period from October 2020 to January 2021. Due to the coronavirus pandemic, the intervention in the homes was suspended for approximately 2 weeks.

Written informed consent was obtained in advance from the legal representatives of the participants. In addition, at the first meeting, participants were also informed in detail about the purpose of the study.

### Sample description

**Fig. 1:**
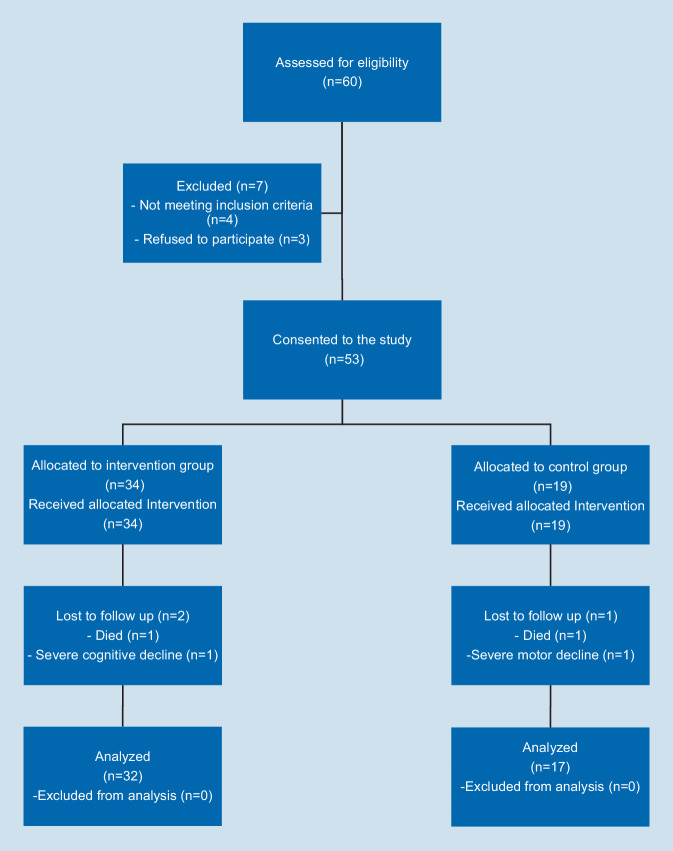
Trial flowchart

Potential subjects were recruited in October 2020 in a nursing home and a dementia center in Magdeburg. Additional dementia facilities in Magdeburg were contacted, unfortunately, there was either no response, or there was no interest. The nurses recruited the subjects of the two inpatient facilities in Magdeburg since they know the potential participants better. There are many relatives of the residents who reject any physical activity of their relatives. The nurses know the relatives and can therefore make a preliminary selection. In selecting potential participants, caregivers were guided by the following inclusion criteria: Participants were included if they were older than 70 years, had mild to moderate dementia, were able to follow a physical activity program, and were able to get around on their own or with a walker. Exclusion criteria were hypertension, severe cardiovascular disease such as cardiac arrhythmias, renal insufficiency, and severe motor disorders. Sixty subjects (53 women/7 men) with dementia participated in the study. Six dropped out again for various reasons (Fig. 1).

Fifty-three subjects (48 women/5 men) were then randomized using block randomization with unequal group sizes (Schulz & Grimes, 2007). This form of randomization was chosen in consultation with the ethics committee because many participants were to be made eligible for the intervention and were not to be denied it. Because of the maximum group size of 8 participants, which allows optimal supervision by 2 test administrators, 34 were assigned to IG (83.91 ± 5.73 years) and 19 to KG (83.06 ± 6.76 years) (Table 3). The blinded participants were randomized by the exercise instructors and controlled for age, Mini-Mental State Examination, and the opinions of the nursing staff. The health status was assessed utilizing a questionnaire.

### Exercise program

The intervention structure is based on the guidelines of the World Health Organization (World Health Organization, 2017, 2019). Since there were two on-site, only group sizes of 8 patients or less where possible for optimal supervision. Through two exercise instructors, an intervention could be provided twice a week for a duration of 45–60 min over 12 weeks. There were always at least 48 h between the two interventions. The program was conducted at a moderate intensity. This intensity form is also recommended in the guidelines (World Health Organization, 2017, 2019). An observation protocol was prepared for each exercise session to record the feasibility of the exercises on the one hand and irregularities in the sessions on the other hand. The control group did not perform any physical exercises and received the usual treatments. Participants in the control group were asked to perform their daily routines and were instructed not to perform any additional exercise activities.

#### Music

Since music, especially in people with dementia, increases the willingness to participate in sports activities (Cloos, 2014), well-known hit songs were played during the exercise programs. Before the start of the study, various music pieces were tested out in the groups. The modified Observed Emotion Rating Scale was used to evaluate the participants’ emotions and their reactions to single music songs (Lawton, van Haitsma, & Klapper, 1996). The age and musical tastes of the participants were taken into account when selecting the music. The Observed Emotion Rating Scale was used for music from the 1940s to the 1980s, and classic genres. In addition to the emotions, the tempo of the music was also crucial, as the music was to be used for training control in the interventions. For this purpose the ranges of 60–80 beats-per-minute (bpm), 81–100 bpm, 101–120 bpm, 121–160 bpm, and > 160 bpm were checked for feasibility. With faster pieces of music from 160 bpm, the tempo had to be adjusted. Based on the findings of the preliminary tests, playlists for the interventions were created that preferably contained music pieces from the 1950s to 1970s, and classics and had a tempo between 60 and 160 bpm (Table 1). The music selected from the genres was mainly from the dementia patients’ youth, as they associated positive emotions with it and remembered the songs. For each unit, a playlist was created with the music tracks and tempo as described above.

**Table 1:** Examples of music tracks

Music title (Interpret)	Beats-per-minute	Beats-per-minute
Die Rosen der Madonna (Bianca)	51	118
Heimwehmelodie (Stefan Mross)	80	130
Schuld war nur der Bossa Nova (Manuela)	83	148
Schöner Fremder Mann (Connie Francis)	85	166
Herzilein (Wildecker Herzbuben)	95	120
Einmal um die ganze Welt (Karel Gott)	105	139

#### Description of intervention

The music-based exercise program was developed as a multidimensional program, with content on strengthening, coordination, balance, as this can be more effective than a single exercise program (Borges-Machado et al., 2020). Since the training was to be carried out with materials available in inpatient care facilities and, due to the mobility restrictions, was to be carried out preferably with the aid of the chair, different emphases were set in the individual lessons, preferably in a sitting position (Cordes et al., 2020). The two exercise sessions per week always had the same topic but differed in the exercises (Table 2). Exercise units were carried out with balls, gymnastic bars, rubber rings, scarves, dice, and units without equipment. The structure of the individual units was always the same. In the beginning, a 10 min warm-up was performed in both sitting and standing positions, followed by a 5 min seated dance routine. The seated dances were changed every 2 weeks to see which ones were feasible and which ones were accepted by the participants. After the dance, the central part of each session began. The main part lasted 30 min and was divided into a coordination part (15 min) and a strengthening part (15 min). In the strength and coordination part, 8–10 exercises were performed. In the coordination exercises, the difficulty level was increased by introducing additional tasks (e.g., handling more than one ball simultaneously) or by adding a secondary cognitive task. In the strength part, the 8–10 exercises should be performed with a repetition count of 8–10. The instructors controlled the execution of the coordination and strengthening exercises. Patients could take breaks whenever they needed them. After that, a final game was played, which lasted 10 min. The final games were performed with the small equipment used to promote interaction and fun among the participants. A 5 min cool-down consisting of stretching exercises and relaxation completed the training. In the training sessions, the music, as described above, was used to increase the frequency and intensity of movements. Since the warm-up, central, and cool-down differed in intensity, the music tempo was also adjusted in each part. Music in the range of 80–120 bpm was played during the warm-up, 60–100 bpm during the central part coordination, 100–160 during the strengthening part, and 60–100 bpm during the ending part. Each training session was supervised by experienced trainers with many years of trained experience in dealing with dementia patients.

**Table 2:** Exercise session (main topics/training targets)

	Exercise session			Exercise session	
Week	Topic	Training targets	Week	Topic	Training targets
Week 1	“Chair fitness with Redondoball”	Coordination, strengthening	Week 7	“Chair gymnastics standing”	Coordination, strengthening, balance
Week 2	“Chair fitness”	Coordination, strengthening, balance	Week 8	“Chair gymnastics with bar + rings”	Coordination, strengthening
Week 3	“Chair gymnastics with bar”	Coordination, strengthening	Week 9	“Chair gymnastics with rings + ball”	Coordination, strengthening
Week 4	“Chair gymnastics with rings”	Coordination, strengthening	Week 10	Gait training	Coordination, strengthening, balance
Week 5	“Chair gymnastics with little bags”	Coordination, strengthening	Week 11	Partner exercises and group games without equipment	Coordination, strengthening, balance, interaction
Week 6	“Chair gymnastics with Thera-Band”	Strengthening	Week 12	Partner exercises and group games with equipment	Coordination, strengthening, balance, interaction

### Instruments and procedure

The primary endpoint was to examine feasibility (Observational protocol) and motor performance, which are indicators of maintaining independence, functional mobility, and fall risk. For this purpose, motor reaction time (drop bar test), grip strength (hand dynamometer test), balance ability (FICSIT-4), mobility, and fall risk (Timed-Up-and-Go test/modified chair rising test) were tested. The secondary endpoints of the study consisted of verbal production ability (verbal fluency “animals”), general cognitive functioning level (MMSE), verbal memory (word list), and attention (Trail Making Test A), and the Quality of life (Qualidem). A detailed description of the Test procedure and Quality criteria is attached to the online supplementary information 1.

#### Feasibility outcomes

To assess the feasibility of the exercises for people with dementia, notes were taken during the training session on which exercises the participants were able to perform. Furthermore, an Observational protocol was used to record general occurrences during the interventions. The two exercise instructors carried out the observations. The comments on the feasibility of the exercises were carried out after each part by both test leaders. After each intervention unit, the intervention in general and the individual exercises were discussed and re-evaluated by both test leaders. In addition, the acceptance of the intervention by the dementia patients was investigated. This was done using the Dementia Mood Picture test (Tappen & Barry, 1995).

#### Motor performance

The data collection of the motor performance always started with the hand dynamometer test (handdy) to determine grip strength (Richards & Palmiter-Thomas, 1996). The hand dynamometer test was performed three times with each subject using both hands. The best value of each hand was included in the evaluation. This was followed by the drop bar test to determine motor reaction time (Fetz & Kornexl, 1978). The drop bar test was performed three times with the previously determined dominant hand with each test person. The best value is included in the evaluation. The modified chair rising test followed this to evaluate the strength ability of the lower extremities (Le Berre et al., 2016). In the modified Chair Rising test (mCRT), subjects must stand up and sit down five times as quickly as possible, but the arms are allowed. The time required is measured. After that, the FICSIT‑4, to assess balance, was performed (Rossiter-Fornoff, Wolf, Wolfson, & Buchner, 1995). The FICSIT‑4 consists of four stances (bilateral stance, semi-tandem stance, tandem stance, and unilateral stance) performed with eyes open and eyes closed. Time is measured and converted to a point scale (maximum 28 points). Finally, the Timed Up and Go test (TUaG) was performed to record mobility (Podsiadlo & Richardson, 1991). The Timed Up and Go test required subjects to stand up from a chair, walk 3 meters, make a 180° turn, walk back, and sit down again. Time was measured in terms of how quickly this was done. In the modified Chair Rising test, the drop bar test, and the Timed Up and Go test, lower values correspond to better performance, whereas higher values in the hand dynamometer test and FICSIT‑4 indicate better performance.

#### Cognitive performance

For the survey of cognitive abilities, the Cerad-NP-Plus (Consortium to Establish a Registry for Alzheimer’s Disease) was conducted (Morris et al., 1989). This questionnaire was developed specifically for dementia patients. Since cognitive abilities counted as a secondary outcome in the study, only five of the ten subtests were included in the final evaluation. These provide initial information about the impact of the program on cognitive abilities. To cover a relatively broad spectrum, the following subtests were included in the analysis: verbal fluency (‘animals’), general cognitive abilities (Mini-Mental State Examination [MMSE]), memory (Word List Saving), and Attention (Trail Making Test A). The Word List Saving is composed of the subtests word list copy and recall. However, in Trail Making Test A, a lower score corresponds to better performance. The other subtests: Boston naming test, constructive practice copy/recall, Word list Recognition recall and Trail Making Test B, were also collected but were not included in the final analysis. The results of these subtests can be found in the online supplementary information 2.

#### Questionnaire for quality of life

The quality of life (QoL) status was screened using the Qualidem (Ettema, Dröes, de Lange, Mellenbergh, & Ribbe, 2007). The questionnaire was developed especially for dementia patients and consists of nine dimensions: care relationship, positive affect, negative affect, restless, tense behavior, positive self-image, social relations, social isolation, feeling at home, and have something to do. The higher the value, the higher the QoL of the person with dementia.

### Statistical data analysis

Due to the small number of subjects, outliers, and the difference in group size, we used nonparametric tests for all statistical analyses (Nahm, 2016) (see online supplementary information 3). Group differences in baseline examination (age, height, weight, body mass index [BMI]) and changes in outcome parameters (mCRT, hand dynamometer test, drop bar test, FICSIT‑4, TUaG, verbal fluency, MMSE, word list retention, Trail Making Test A, Qualidem) were compared with the Mann–Whitney U test. Changes between measurement time points within a group were tested with the Wilcoxon test (time effect). Outcome parameters are presented at the median (25th/75th percentile). All statistical analyses were performed with SPSS Statistics 26 (IBM, Inc., Chicago, IL, USA). The significance level was set at an alpha (α) level of 5%. Observational protocol was evaluated qualitatively.

## Results

### Observational protocol

The Dementia Mood Picture test showed that the subjects were more likely to be in a good mood after the interventions than before. Before the interventions, 15% of the subjects were in a bad mood or felt anxious. After the intervention, this number was reduced to 5%. Concerning the feasibility of the exercises, 85% of all exercises could be performed by the subjects. When completing the exercises, however, the different execution of the participants has been noticeable. For example, some were able to perform almost all of the exercises without difficulty, while some had difficulty performing individual exercises. This was due to the different degrees of dementia in the groups. Participants with mild dementia were able to perform and understand the exercises better than participants with moderate or severe dementia. Groups should therefore be formed with uniform degrees of dementia to ensure optimal intensity. The observation protocol also revealed that a loud voice, clearly understandable instructions, and dry runs were necessary to execute the exercises better. The training should also be modular. This means that before playing the music, the exercises should be demonstrated first, otherwise, the subjects will concentrate on the music rather than the exercises. The music served as anchor points and improved the mood of the dementia patients during the sessions. However, the beat of the music was better perceived with speech and signal tones (e.g., clapping). From the observation protocol, it could also be determined that experiences of success must be pointed out and named. Compliments, recognition, and individual attention to the respective persons are decisive for dementia patients and motivate them. In addition, the observation sheets showed that the interactive exercises and games were particularly popular with the participants. The games mainly promote interaction and the dynamics of the group. The larger the group, the greater the interaction and dynamic effect. For a larger group composition, more trainers are needed to ensure optimal support.

### Motor performance, cognition, and quality of life

The data of four subjects had to be excluded. Two subjects, each from the intervention and control group, dropped out within the 12 weeks. The reasons were cognitive and motor decline or death of the participants. Finally, data from 49 subjects (intervention group *n* = 32; control group *n* = 17) were analyzed (see flowchart in Fig. 1). At baseline, age, height, weight, and BMI analysis revealed no significant differences between groups (Table 3). A detailed sample characterization can be found in online supplementary information 4.

**Table 3:** Sample characteristics (mean ± SD)

Baseline characteristic	Intervention group (*n* = 32)	Control group (*n* = 17)	*p‑Value*
Age (years, mean ± SD)	83.91 ± 5.73	83.06 ± 6.76	0.789
Body mass index (kg/m^2^, mean ± SD)	27.07 ± 5.71	28.08 ± 4.68	0.433
Sex (%)	m: 15.2% f: 84.8%	m: 12.5% f: 87.5%	0.804
MMSE (score, mean ± SD)	16.42 ± 6.71	18.59 ± 6.83	0.380

*MMSE* Mini-Mental State Examination, *SD* standard deviation

*p < 0.05

To begin with, it was shown that all motor and cognitive test procedures performed could be carried out without any major problems. The only point to note is the performance of the Trail Making Test B, which could only be performed by a few subjects due to its complexity and was thus not shown to be suitable for dementia patients.

Regarding the motor performance assessed, we found no statistically significant differences between the groups in the pretest (mCRT: Z = −0.588, *p* = 0.577; drop bar test: Z = −0.582, *p* = 0.560; hand dynamometer right/left: Z = −0.221, *p* = 0.825 and Z = −0.074, *p* = 0.941, respectively; FICSIT-4: Z = −0.810, *p* = 0.418; TUaG: Z = −1.006, *p* = 0.314). The differences between the groups regarding the hand dynamometer right/left, and drop bar test were also not statistically significant at posttest (Z = −1.313, *p* = 0.189 and Z = −0.767, *p* = 0.443 and Z = −0.875, *p* = 0.382, respectively). In contrast, the modified Chair Rising test (mCRT), FICSIT‑4 and Timed Up and Go test (TUaG) showed significant differences between groups in the posttest (Z = −2.133, *p* = 0.033; Z = −2.104, *p* = 0.035; Z = −2.822, *p* = 0.005; Table 4). Statistically significant time effects also occurred in the intervention group. Participants in the intervention significantly improved over 12 weeks in mCRT, left hand dynamometer and TUaG (Table 4). In contrast, a significant worsening of the control group in balance ability was observed (Table 4). Looking at the differences from pretest to posttest within each group, it can be seen that the participants in the intervention group reduced their time to perform the TUaG or the mCRT by 3–4 s or improved in grip strength by 20 N, while the values in the control group either remained the same or worsened (Table 4). For verbal fluency, MMSE, Word List Saving, Trail Making Test A, and Qualidem, we were unable to show statistically significant differences between the intervention group and control group in the pretests (verbal fluency: 10.0 [6.25/14.0] and 9.0 [5.50/15.0], Z = −0.032, *p* = 0.975; MMSE: 17.0 [11.25/22.0] and 20.0 [12.50/25.50], Z = −0.936, *p* = 0.349; Word List Saving: 0.00 [0.00/50.00] and 40.00 [0.00/63.33], respectively, Z = −1.394, *p* = 0.163; TMT-A: 175.50 [100.50/235.50] and 129.50 [84.25/251.25], Z = −0.780, *p* = 0.435; Qualidem: 182.00 [177.00/208.25] and 192.00 [167.50/207.50], Z = −0.242, *p* = 0.809). No significant differences between groups were observed after the 12-week intervention (verbal fluency: Z = −1.442, *p* = 0.149; MMSE: Z = −0.021, *p* = 0.983; Word List Saving: Z = −0.577, *p* = 0.564; TMT-A: Z = −0.137, *p* = 0.891; Qualidem: Z = −0.206, *p* = 0.842). Regarding verbal fluency and Trail Making Test A, a significant time effect was observed in the intervention group, showing that after 12 weeks of intervention, subjects could name more terms in 1 min (+2.0 [0.00/2.75]) and perform faster the task from Trail Making Test A (−14[−33.25/12.50]). In contrast, subjects in the control group deteriorated significantly over time in verbal fluency (−2.0 [2.00/−3.00]) and additionally in general cognitive ability (MMSE) (−6 [1.00/−7.00]). In the other cognitive test procedures and quality of life, we did not detect any time effects in either group (Table 4; online supplementary information 2).

**Table 4:** Medians and 25th/75th percentiles of the mobility and fall risk (modified Chair Rising test, Timed Up and Go test), hand grip strength, balance (FICSIT-4), motor reaction speed (drop bar test), verbal fluency, general cognitive ability (MMST), memory (Wordlist Saving), attention (Trail Making Test A), and quality of life (Qualidem) for the intervention group, and control group in the pre-and posttest

Test procedure	Intervention group *n* = 32			Control group *n* = 17		
	Pretest Median (25th/75th percentile)	Posttest Median (25th/75th percentile)	Wilcoxon test (z-value; *p*-value)	Pretest Median (25th/75th percentile)	Posttest Median (25th/75th percentile)	Wilcoxon test (z-value; *p*-value)
*Motor performance*						
Hand grip strength right (N)	134.39 (96.14/177.81)	153.53 (96.88/194.73)	Z = −1.460 *p* = 0.144	144.21 (84.37/173.64)	139.30 (86.33/158.43)	Z = −0.781 *p* = 0.435
Hand grip strength left (N)	116.86 (88.29/160.39)	141.76 (98.35/176.83)	Z = −3.366 *p* = 0.001	124.59 (75.04/162.36)	129.49 (94.18/164.32)	Z = −0.310 *p* = 0.756
Drop bar test (cm)	28.00 (18.00/36.00)	24.00 (16.00/32.00)	Z = −2.131 *p* =0.033	24.50 (16.25/33.00)	26.00 (18.00/40.75)	Z = −1.620 *p* = 0.105
Modified chair rising test (s)	17.60 (12.78/22.48)	14.60 (11.98/18.88)^a^	Z = −4.357 *p* < 0.001	19.15 (13.13/28.70)	17.95 (14.55/25.23)	Z = −0.310 *p* = 0.756
FICSIT‑4 (s)	15.00 (9.00/23.00)	16.00^a^ (9.50/23.00)	Z = −1.134 *p* = 0.257	16.00 (5.50/19.50)	13.00 (3.00/16.00)	Z = −3.221 *p* = 0.001
Timed Up and Go test (s)	19.25 (12.38/33.45)	13.50^a^ (9.63/26.48)	Z = −3.929 *p* < 0.001	21.25 (18.88/25.85)	24.45 (18.73/28.76)	Z = −0.879 *p* = 0.379
*Cognition performance*						
Verbal fluency (max. number)	10.00 (6.25/14.00)	12.00 (9.25/15.75)	Z = −3.030 *p* = 0.002	9.00 (5.50/15.00)	7.00 (5.50/14.50)	Z = −2.342 *p* = 0.019
MMSE (total points)	17.00 (11.25/22.00)	17.50 (12.25/21.75)	Z = −0.178 *p* = 0.859	20.00 (12.50/25.50)	14.00 (12.00/23.50)	Z = −2.891 *p* = 0.004
Wordlist Saving (%)	0.00 (0.00/50.00)	12.50 (0.00/66.67)	Z = −1.043 *p* = 0.297	40.00 (0.00/63.33)	33.33 (0.00/55.00)	Z = −0.756 *p* = 0.449
Trail Making Test A (s)	175.50 (100.50/235.50)	161.50 (100.75/221.25)	Z = −2.058 *p* = 0.040	129.50 (84.25/251.25)	123.50 (84.50/272.25)	Z = −1.818 *p* = 0.069
*Questionnaires*						
Qualidem (Total points max. 240)	182.00 (177.00/208.25)	190.50 (167.50/207.00)	Z = −0.284 *p* = 0.776	192.00 (167.50/207.50)	200.00 (176.50/205.50)	Z = −0.280 *p* = 0.977

*MMSE* Mini-Mental State Examination. *FICSIT‑4* Frailty and Injuries: Cooperative Studies of Intervention Techniques

^a^ Group effect: significantly different from the control group at posttest (statistically significant: *p* ≤ 0.05)

## Discussion

Experiencing a pleasant old age is the life goal of every person, and it is necessary to make this possible. However, in particular, for people with dementia, this is not always possible due to the disease, as the risk of falling is increased, and thus their independence is limited. It is assumed that through the combination of music and physical activity, a stabilization of the cognitive and motor performance of people with dementia can be achieved, as these abilities are receptive through music or physical activity until the late phase. Especially the preservation of motor performance is necessary for maintaining independence, which, however, is influenced by cognitive performance. Therefore, this study aimed to investigate the feasibility influence of a multidimensional music- and chair-based physical activity program on selected motor and individual cognitive performance in dementia patients. In the beginning, it could be shown that this form of intervention could be carried out with dementia patients and was positively accepted by them. In addition, this pilot study shows initial trends that a multimodal approach consisting of training multiple skills and abilities can improve motor performance and selected cognitive performance compared to no intervention. These results are congruent with other similar studies. The results of Borges-Machado et al. (2020) also confirmed that a multidimensional exercise program can be implemented in dementia patients and has positive effects. In this study, however, it was also shown that the factor music could also be integrated and used to increase motivation and intensity. Likewise, the results could show the positive effects of a chair-based intervention, thus confirming the results of Cordes et al. (2020, 2021) who had investigated this form of intervention in the nursing setting. After elastic band training, Chen et al. (2016) examined handgrip strength in older adults with cognitive impairment and found significant improvement. Hand strength is an indicator of well-being and overall strength (Taekema, Gussekloo, Maier, Westendorp, & de Craen, 2010) and increased after music-based training. Blankevoort et al. (2010) demonstrated that lower limb strength improved equally with multicomponent interventions and progressive resistance training. In this study, lower limb strength also improved (modified Chair Rising test), which could be due to moderate training volume. Blankevoort et al. (2010) additionally found a similar effect on mobility (Timed Up and Go test), which also improved in this study after the 12-week intervention. In addition, we observed group effects indicating that mobility and balance significantly improved in the intervention group compared with the control group at the end of the intervention (Table 4).

In the cognitive domain, significant improvements were found in verbal fluency and executive function. Brancatisano et al. (2019) and van de Winckel, Feys, de Weerdt, and Dom (2004) found similar effects following their music-based exercise programs. They also demonstrated significant improvement in MMSE scores. The present study results could only descriptively show a slight improvement in MMSE score after the intervention. Cognitive performance improvement may also be since many exercises in the program also included motor and cognitive tasks, which may positively influence cognitive performance (Northey, Cherbuin, Pumpa, Smee, & Rattray, 2018).

On the other hand, significant deterioration in verbal fluency, general cognitive abilities, and balance was found in the control group. Similar results were obtained by Toulotte, Fabre, Dangremont, Lensel, and Thévenon (2003). They demonstrated that people with dementia experience a loss of function within three months, even without intervention.

Despite the positive effects of the intervention, quality of life scores did not improve after the intervention. Accordingly, quality of life (QoL) did not change within the study period, which is also consistent with the work of Ojagbemi and Akin-Ojagbemi (2019). Quality of life in people with dementia is a complex parameter to measure. It is known that QoL assessments performed by proxies are influenced by the level of distress or the state of the emotional well-being of the rater (Conde-Sala, Garre-Olmo, Turró-Garriga, López-Pousa, & Vilalta-Franch, 2009).

In addition to physical activity, music also had an impact on outcomes. People with dementia are receptive to music, positively influencing their mood and behavior (Cloos, 2014). However, it should be noted that music can only positively affect if it is familiar and perceived as pleasant (Gembris, 2009; Gómez Gallego & Gómez García, 2017). Music is perceived very individually and can just as quickly have adverse effects on dementia patients if it is perceived as disturbing (Gómez Gallego & Gómez García, 2017). Therefore, each subject should be allowed to listen to his or her music, but this is not easy to do in a group training session. However, exercise training should promote interaction and social components not to isolate patients (DGPPN, 2017). An additional factor that could have led to the positive effects is the communication used during the intervention. In dementia patients, communication takes on great importance and can create a feel-good atmosphere in which the dementia patients feel more comfortable and perform the exercises better (Eggenberger et al., 2013; van Alphen et al., 2016). This should also be considered in subsequent studies.

Based on these results, our first hypothesis that a piece of multidimensional music and chair-based intervention is feasible and can improve motor function can be accepted. The second hypothesis that the intervention can improve cognition and quality of life can also be accepted, as cognition and quality of life either improved or remained stable during the intervention, which can be considered positive in dementia patients.

## Limitations

Some limitations need to be considered for future studies. The multimodal approach should be applied to larger cohorts to consolidate results. Block randomization with groups of equal size would be preferable in the following full study since the better statistical discriminatory power would allow more accurate conclusions in this case. The Dementia Mood Picture test is not appropriate for use in moderate to severe dementia (Oppikofer, 2008).

Furthermore, no total score should be calculated for all subscales in the Qualidem. However, this was done for methodological and statistical reasons (Dichter et al., 2015). Likewise, when surveying quality of life, caregivers should be blinded in following studies. Quality of life is mainly assessed by questionnaires that are subjectively rated by the caregivers. In this respect, the data could be falsified if you know who is in which group. The duration and the intensity of the training programs would need to be differentiated for dementia levels to avoid too high or too low a burden for patients (Uijen, Aaronson, Karssemeijer, Rikkert, & Kessels, 2020). In addition to dividing the groups according to the degree of dementia, the size of the group also plays a decisive role. As already mentioned, the size of the group is a crucial factor for interaction and dynamics. The exercise program should be tested with larger groups in follow-up studies to allow greater interaction and dynamic effect. It has to be taken into account that more support staff is needed to implement the program in this respect. In addition to considering the degree of dementia and group size, group composition and homogeneity must also be considered in the subsequent studies. In this study, for example, over 80% of the participants were female. In order to make generally valid statements, more male dementia patients must be included. Furthermore, future studies should examine not only statistical significance but also clinical relevance. (Wink, 2018). Then, conclusions could also be made about the effectiveness of the exercise program, especially for everyday life. Likewise, cognitive and dual-task tasks should also be incorporated into such an exercise program. This is particularly useful for fall prevention, as limited cognitive abilities result in insufficient compensation for sensory dysfunctions, thus increasing the risk of falls (Hamacher, Hamacher, & Schega, 2014). Furthermore, the inclusion and exclusion criteria should be refined. Attention must be paid to hearing ability since hearing and feeling the music is crucial in music-based programs. However, especially in this age structure and with dementia patients, hearing ability is restricted, so that one must find alternatives so that all can hear and feel the music. However, the duration of the intervention and the number of training sessions should also be considered. Due to organizational reasons, the intervention of this study could be performed two times a week for 60 min over 12 weeks. In guidelines, a longer intervention time and more frequent training sessions are recommended for lasting effects (World Health Organization, 2019).

## Conclusion

It was shown that a piece of multidimensional music- and chair-based exercise program for dementia patients targeting strength, endurance, coordination, and balance was developed and tested. The exercise program was well accepted by the patients and also showed isolated positive results. In addition to the isolated positive results, no significant deterioration was found in any of the tested test procedures, and thus stabilization or resource preservation was achieved. These results are also to be considered positive in the case of dementia. However, the effects were too small to be considered clinically significant, mainly due to the small cohorts. However, the results suggest that the applied intervention may improve parameters relevant to maintaining independence more sustainably than usual care. These results suggest that a subsequent randomized, controlled trial (RCT) would be useful. In doing so, the limitations identified in this study have to considered and adjusted.

After conducting an RCT based on this study, it could be shown that a piece of multidimensional music- and chair-based exercise program can serve as a nondrug alternative or adjunct to drug therapies.

## Supplementary Information

1. Test procedures

2. Results of the additional subtests

3. Box plots

4. Sample characteristics

## Declarations

All procedures performed in studies involving human participants or on human tissue were in accordance with the ethical standards of the institutional and/or national research committee and with the 1975 Helsinki declaration and its later amendments or comparable ethical standards. Approval was granted by the Ethics Committee of University Magdeburg (No. 100/20). Trial registration number (DRKS00021170) and date of registration (03 August 2020). Informed consent was obtained from all individual participants included in the study.
