# Supplementary material for: Influence of a multidimensional music-based exercise program on selected cognitive and motor skills in dementia patients—a pilot study
Source: Ger J Exerc Sport Res. 2021 Oct 15;51(4):495–505. doi: 10.1007/s12662-021-00765-z (PMC8519499; doi:10.1007/s12662-021-00765-z)
Supplement: Supplementary file 1 — 1. Test procedures [file 12662_2021_765_MOESM1_ESM.pdf]

Influence of a multidimensional music-based exercise program on selected cognitive and motor skills in dementia patients – A Pilot-Study.

Prinz A.[1], Schumacher A.[1], Witte K.[1]

1 Department of Sport Science, Otto-von-Guericke University Magdeburg, Magdeburg, Germany

Corresponding author

Alexander Prinz M.Sc.

Zschokkestr. 32

39104 Magdeburg

0391/67-54862

alexander.prinz@ovgu.de

ORCID: 0000-0003-4416-7913

Author contributions

All authors contributed to the study conception and design. Material preparation, data collection and analysis were performed by [Prinz A.] and [Schumacher A.]. The first draft of the manuscript was written by [Prinz A.] and all authors commented on previous versions of the manuscript. All authors read and approved the final manuscript.

Table 1 cognitive test procedures

cognition		
**CERAD-NP-Plus** (Morris et al. 1989)		
**Test procedure (subtest)**	**Cognitive area**	**Declaration**
verbal fluency (animals)	Executive functions	- - name as many animals as possible in one minute<br>- - Score is unlimited
Boston-Naming-Test	Word finding and naming, visual perception	- the short form contains 15 images that would need to be named<br>- 3 groups of items:<br>o first group contains 5 frequent items<br>o the second group contains 5 medium frequent items,<br>o the third group contains 5 rare items
Mini-Mental State Examination	General cognitive function level (Screening)	- Answering questions to determine the cognitive abilities of older people<br>- - maximum 30 points
Memory list sum of immediate recall	Verbal memory	- Ten words are read out one after the other and then recalled from memory.<br>- - is performed three times, are the same ten terms only in different orders<br>- - Number of remembered terms in the three rounds á ten terms (max. 30 points possible)
Constructional praxis copy	Visuoconstructive skills	- 4 pictures have to be painted (circle, parallelogram, 2-square, cube)<br>- - maximum 11 points
Word list delayed recall	Verbal memory (delayed verbal memory)	- free reproduction of the 10 terms from the word list memory test<br>- - maximum 10 points
Word list Recognition recall	Verbal memory (delayed verbal memory, recognition)	- - the 10 already mentioned terms and 10 new terms are presented to the respondent<br>- - the respondent has to declare the 10 already mentioned terms as "already known" and the 10 new ones as "not known".

Constructional praxis recall	Nonverbal memory (delayed figural memory)	- Drawing the figures from the constructive part from memory<br>- - maximum 11 points
Trail-Making-Test A	Psychomotor speed,<br><br>Executive functions	- number linking as fast as possible (from 1-25)<br>- - max. 180 s
Trail-Making-<br><br>Test B	Executive functions	- combine numbers and letters alternately, keeping the numerical and alphabetical sequence, respectively (1-13; A-L)<br>- max. 300 s
Quality criteria:<br><br>- Reliability: Interrater reliability: ICC = 0.92 - 1.00 (American version)<br>- Test-restest reliability: good according to authors<br>-<br>- Validity:<br>o Criterion validity: for American version moderate to high correlation with other dementia measures (Ex. R = -.83 Clinical Dementia Rating Scale).<br>o Construct validity: sensitive differentiation between healthy and dementia patients and between the severity of cognitive impairment.		

Table 2 motor skills test procedure

Motor function		
**Test procedure**	**Motor area**	**declaration**
modified Chair-Rising-Test<br><br>(Le Berre et al. 2016)	Strength and coordination of the leg muscles	- Measures how long it takes a respondent to stand up and sit down five times with hand assistance.<br>- Score from zero (>60s, high risk of falling) to four (<11.2s, low risk of falling)
	Quality criteria:<br><br>• Reliability: Interrater reliability: ICC = 0.99 - 1.00<br>o Test-retest reliability: ICC = 0.95 - 0.99<br><br>• Validity:<br>o Construct validity: strong convergences with physical performance (r = -0.59; Incremental Shuttle Walk Test).	

Drop bar-Test<br><br>(Fetz et al. 1978)	Elementary (simple) reactivity	- Response measured how fast the subject can grasp the falling rod (in cm)<br>- subject has three repetitions with the dominant hand<br>- best value goes into the evaluation
	Quality criteria:<br><br>• Objectivity: .83 - .95 (Fetz & Konexl, 1978, p.52); .72 - .91 (Richter & Beuker, 1976).<br>• Reliability: .83 - .91 (Fetz & Konexl, 1978, p.52); .58 (Richter & Beuker, 1976)	
Hand Dynamometer<br><br>(Richards et al. 1996)	Hand force<br>(current muscle force)	- grip strength is measured in both hands (in Newton)<br>- three repetitions are performed with each hand<br>- maximum value is included in the evaluation
	Quality criteria:<br><br>• Objectivity very high if the exact implementation rules are followed (Oltman, 2012).<br>• Reliability: .89 -.96 (Fetz & Kornexl, 1993)<br>• Content- and construct-related validity is taken as given (Oltmann, 2012)<br>• Criterion-related validity is also taken as given (Oltmann, 2012).	
Timed-Up-and-Go-Test<br><br>D. Podsiadlo & S. Richardson (1991)	Mobility restriction	The test person is to stand up from a sitting position without assistance, walk back and forth for 3 meters and sit down again (aids are allowed)<br><br>Score from ≤10s (Everyday mobility unrestricted) to ≥30 (Pronounced mobility restriction, usually intervention/ Need for assistive devices)
	Quality criteria:<br><br>• Reliability: Interrater reliability: ICC = 0.91 (Rydwik,2011)<br>o Test-retest reliability: ICC = 0.96 - 0.99 (Flansbierr,2005)<br>• Validity:<br>o Criterion validity: Berg Balance Scale (r=0.81), Barthel Index (r=0.78) (Podsiadlo, 1991)	

Table 3 Quality of life

<table>
<tr><th colspan="3">Quality of life</th></tr>
<tr><th>Test procedure</th><th>Area</th><th>declaration</th></tr>
<tr><td>NOSGER II

(Spiegel et al. 1991)</td><td>Quality of life (screening)</td><td>- Questionnaire filled out by the nursing staff (external assessment)
- A total of 30 items distributed over the following dimensions:
o Memory,
o Instrumental activities
o Activities of daily living
o mood
o social behavior
o disruptive behavior
- Response options from "always" to "never
- low values no or only minor disturbances
- high values clear disturbances in the behavioral areas</td></tr>
<tr><td colspan="3">Quality criteria:
• Interrater reliability: r = .53 - .89
• Retest reliability: r = .84 - .92 (2 weeks); r = .75 - .93 (30 days)
• Validity:
o Criterion validity: varying degrees of correlation with external criteria, depending on the dimension considered (ex. MMSE: r = .32 - .82)
o Construct validity: discrimination between healthy and impaired persons with regard to dementia.</td></tr>
</table>
