# Supplementary material for: Influence of a multidimensional music-based exercise program on selected cognitive and motor skills in dementia patients—a pilot study
Source: Ger J Exerc Sport Res. 2021 Oct 15;51(4):495–505. doi: 10.1007/s12662-021-00765-z (PMC8519499; doi:10.1007/s12662-021-00765-z)
Supplement: Supplementary file 2 — 2. Results of the additional subtests [file 12662_2021_765_MOESM2_ESM.pdf]

Influence of a multidimensional music-based exercise program on selected cognitive and motor skills in dementia patients – A Pilot-Study.

Prinz A.[1], Schumacher A.[1], Witte K.[1]
1 Department of Sport Science, Otto-von-Guericke University Magdeburg, Magdeburg, Germany

Corresponding author

Alexander Prinz M.Sc.

Zschokkestr. 32

39104 Magdeburg

0391/67-54862

alexander.prinz@ovgu.de

ORCID: 0000-0003-4416-7913

Author contributions

All authors contributed to the study conception and design. Material preparation, data collection and analysis were performed by [Prinz A.] and [Schumacher A.]. The first draft of the manuscript was written by [Prinz A.] and all authors commented on previous versions of the manuscript. All authors read and approved the final manuscript.

Table 1

Results of the additional subtests of the Cerad-NP-Plus test battery.

Test procedure	Intervention group n=32			Control group n=17		
	Pre-Test Median (25th/75th percentile)	Post-Test Median (25th/75th percentile)	Wilcoxon-Test (Z-Value; p-Value)	Pre-Test Median (25th/75th percentile)	Post-Test Median (25th/75th percentile)	Wilcoxon-Test (Z-Value; p-Value)
Cognition						
Bosten-Naming (max. number)	9.00 (6.00/11.75)	9.00 (7.00/12.00)	Z = -2.928 p = .003	10.00 (5.00/13.50)	10.00 (5.00/12.50)	Z = -1.496 p = .135
Constructive practice copy (total points)	7.00 (4.25/7.00)	7.00 (5.00/7.00)	Z = -0.605 p = .545	6.00 (2.50/7.00)	7.00 (3.50/8.00)	Z = -2.448 p = .014
Constructive practice recall (total points)	0.00 (0.00/0.00)	0.00 (0.00/0.00)	Z = -1.000 p = .317	0.00 (0.00/4.50)	0.00 (0.00/4.00)	Z = -1.473 p = .141
Word list Recognition recall (%)	52.50 (50.00/85.00)	67.50 (50.00/83.75)	Z = -1.467 p = .142	70.00 (50.00/100.00)	75.00 (50.00/85.00)	Z = -0.973 p = .331
Trail-Making-Test-B (s)	291.00 (257.00/373.25)	309.00 (261.00/418.00)	Z = -1.530 p = .126	292.00 (142.00/333.25)	325.00 (162.25/374.00)	Z = -1.826 p = .068
