# Supplementary material for: Influence of a multidimensional music-based exercise program on selected cognitive and motor skills in dementia patients—a pilot study
Source: Ger J Exerc Sport Res. 2021 Oct 15;51(4):495–505. doi: 10.1007/s12662-021-00765-z (PMC8519499; doi:10.1007/s12662-021-00765-z)
Supplement: Supplementary file 3 — 3. Box plots [file 12662_2021_765_MOESM3_ESM.pdf]

Influence of a multidimensional music-based exercise program on selected cognitive and motor skills in dementia patients - A Pilot-Study.

German Journal of Exercise and Sport Research

Prinz A.[1], Schumacher A.[1], Witte K.[1]

1 Department of Sport Science, Otto-von-Guericke University Magdeburg, Magdeburg, Germany

Corresponding author

Alexander Prinz M.Sc.

Zschokkestr. 32

39104 Magdeburg

0391/67-54862

alexander.prinz@ovgu.de

ORCID: 0000-0003-4416-7913

Diagrams

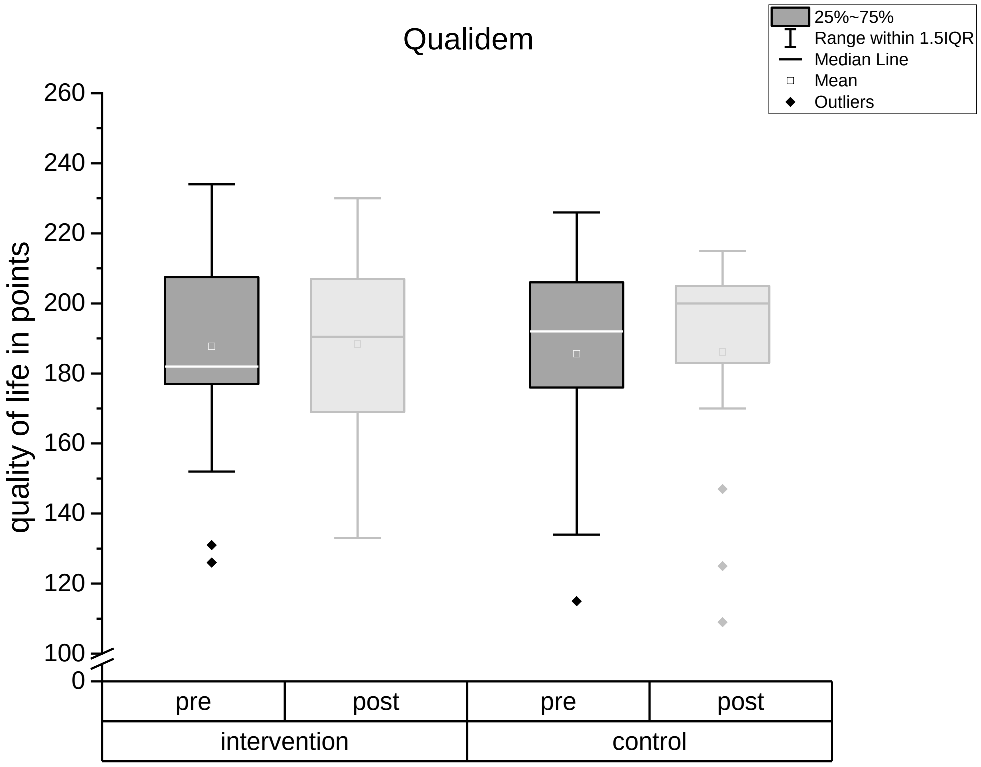

Fig. 1 *Results of the intervention group and control group in the pre- and post-test at the Qualidem*

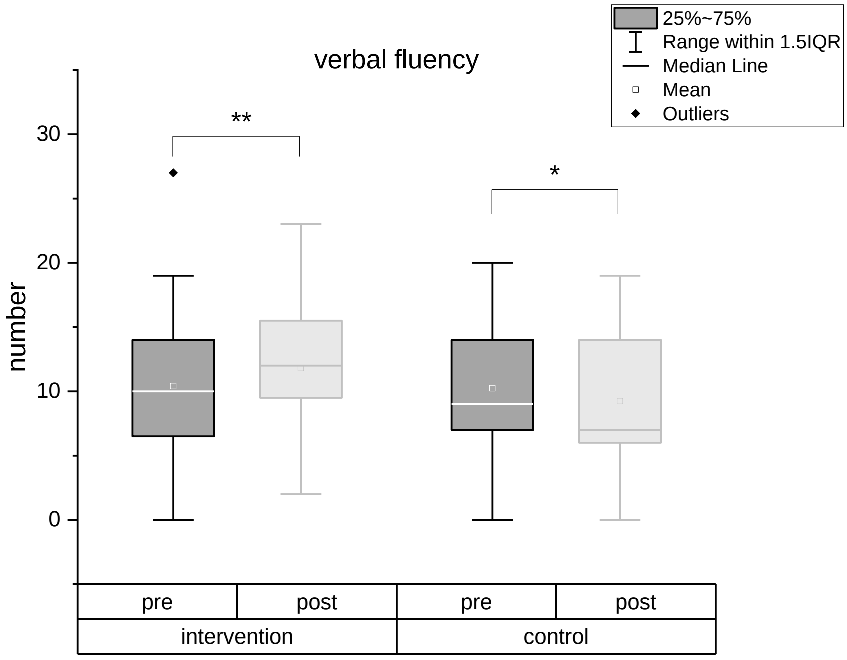

Fig. 2 *Results of the intervention group and control group in the pre- and post-test in verbal fluency.*

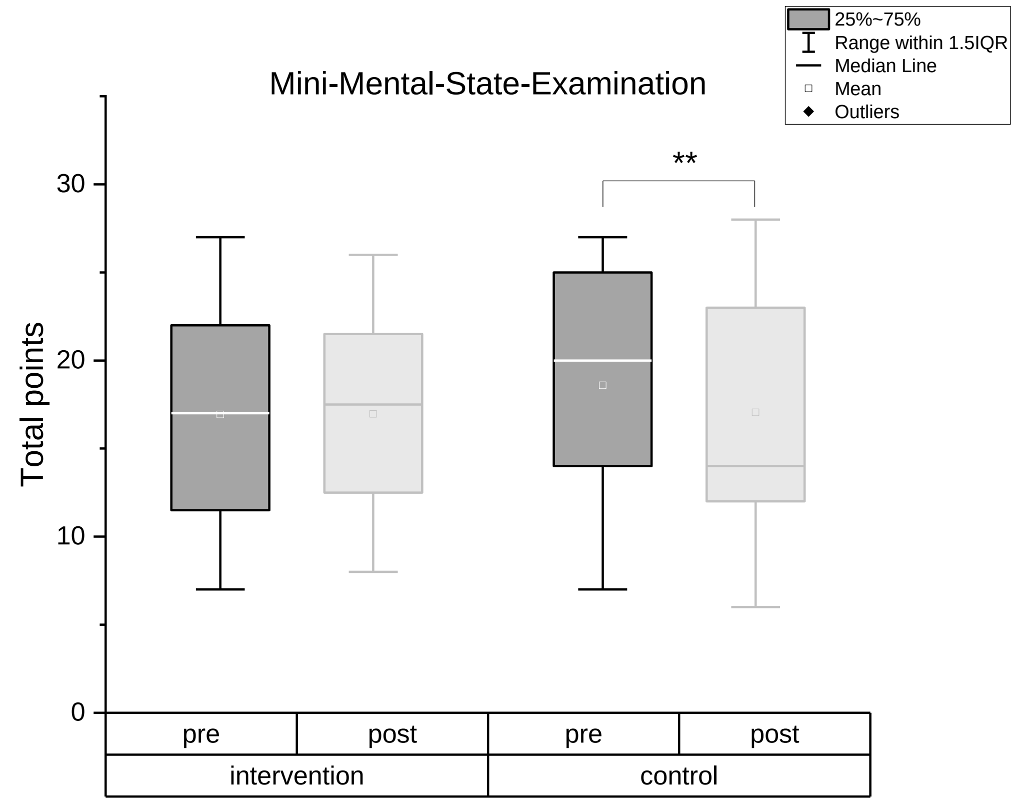

Fig. 3 *Results of the intervention group and control group in the pre- and post-test in the Mini-Mental Status Examination*

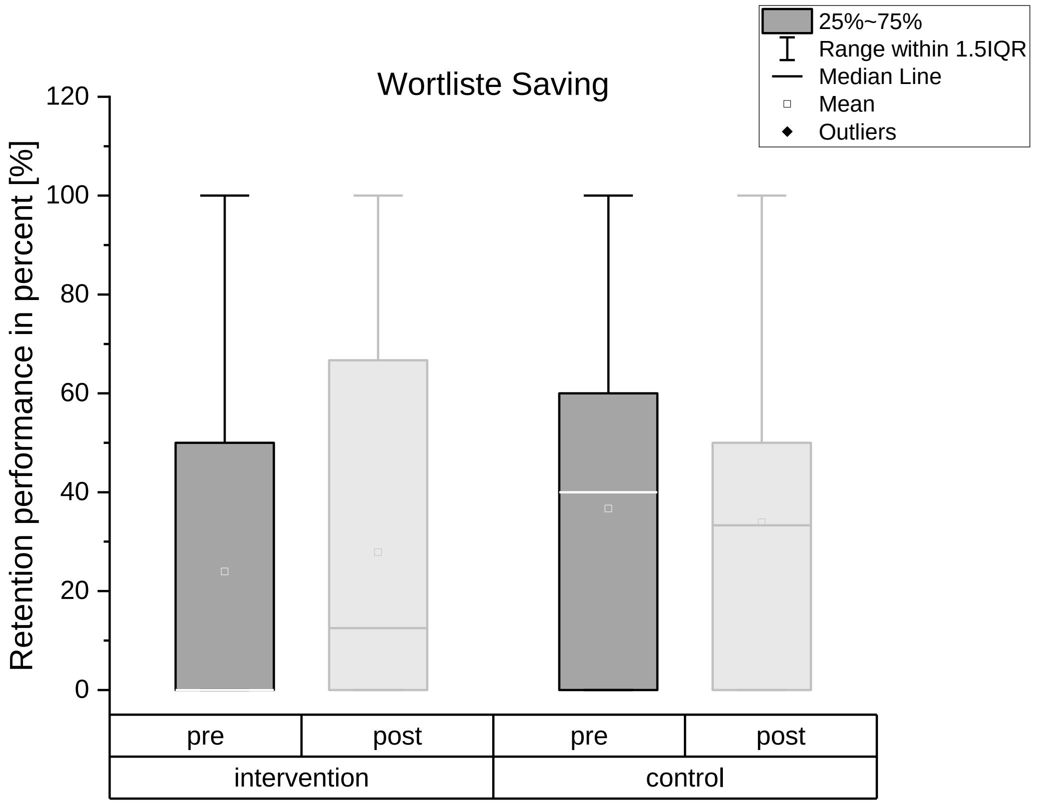

Fig. 4 *Results of the Intervention Group and Control Group in the Pre- and Post-Test in Word List Saving*

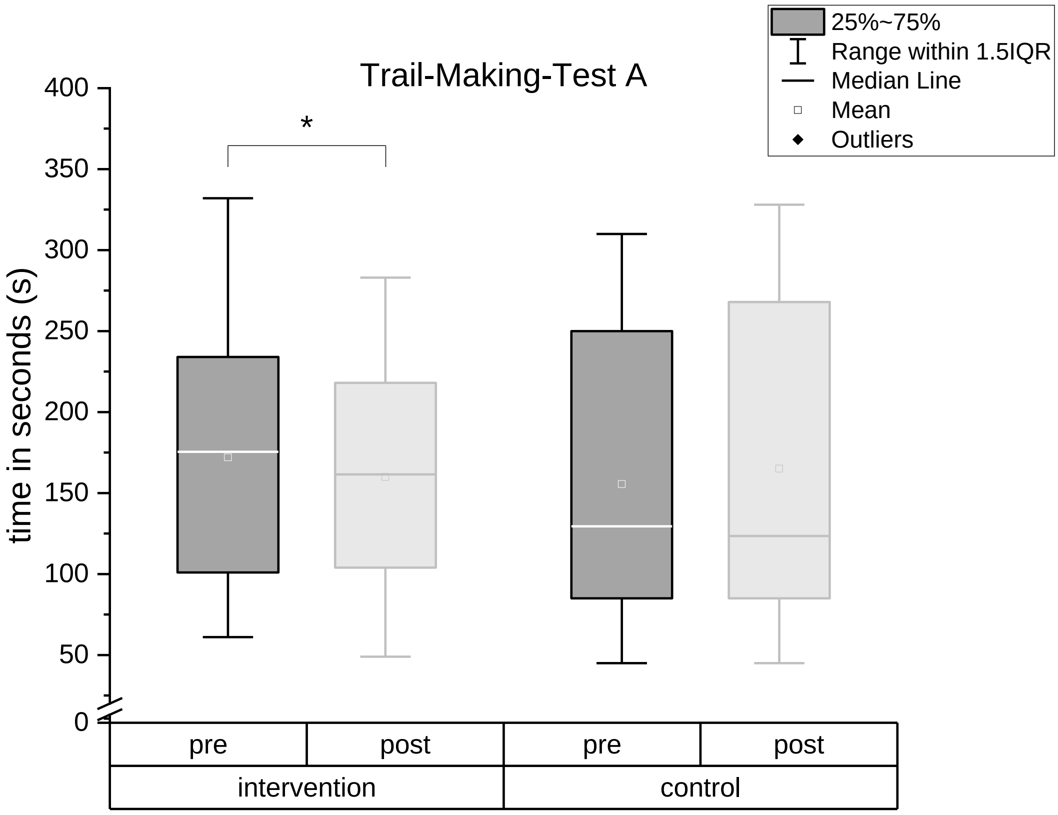

Fig. 5 *Results of the intervention group and control group in the pre- and post-test in the trail making test A*

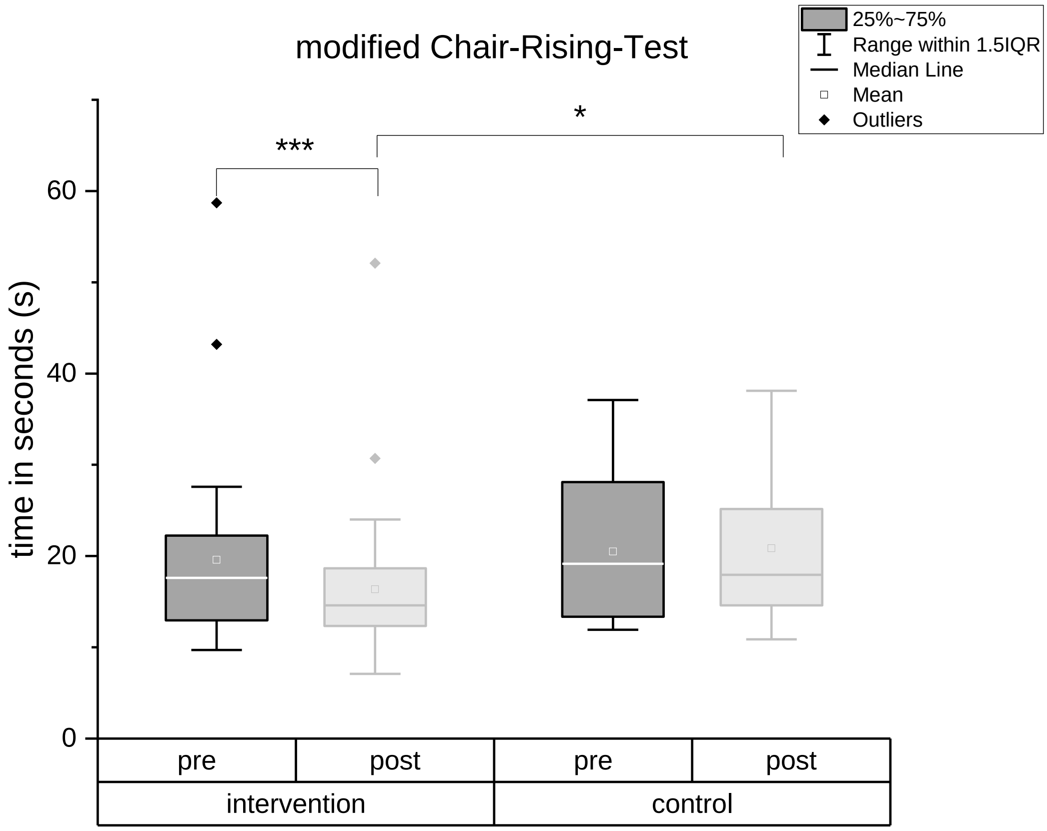

Fig. 6 *Results of the intervention group and control group in the pre- and post-test in the modified chair-rising test*

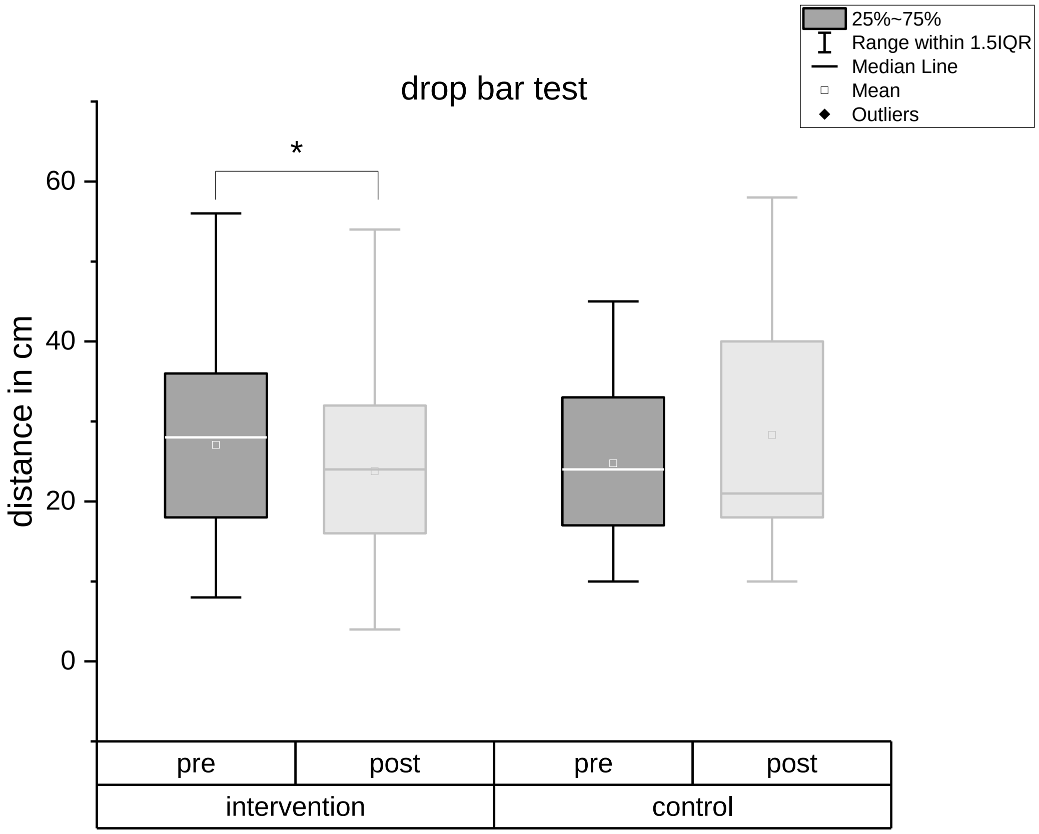

Fig. 7 *Results of the intervention group and control group in the pre- and post-test in the drop bar test*

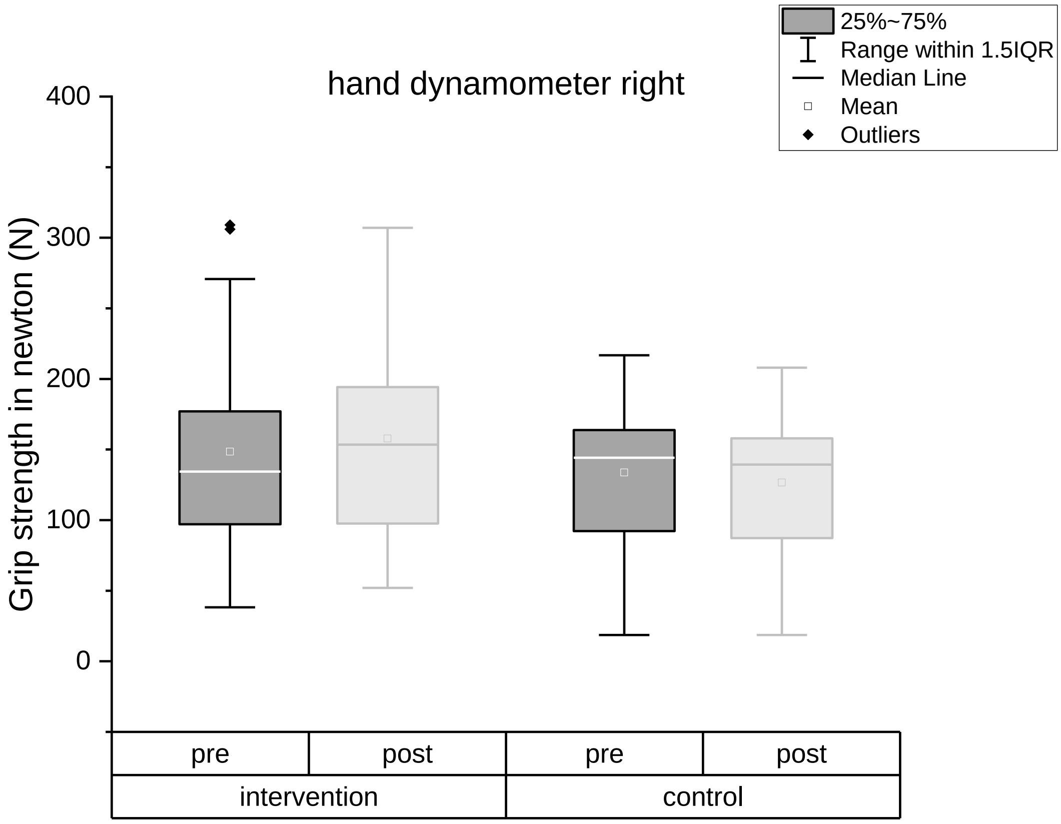

Fig. 8 *Results of the intervention group and control group in the pre- and post-test at the hand dynamometer right*

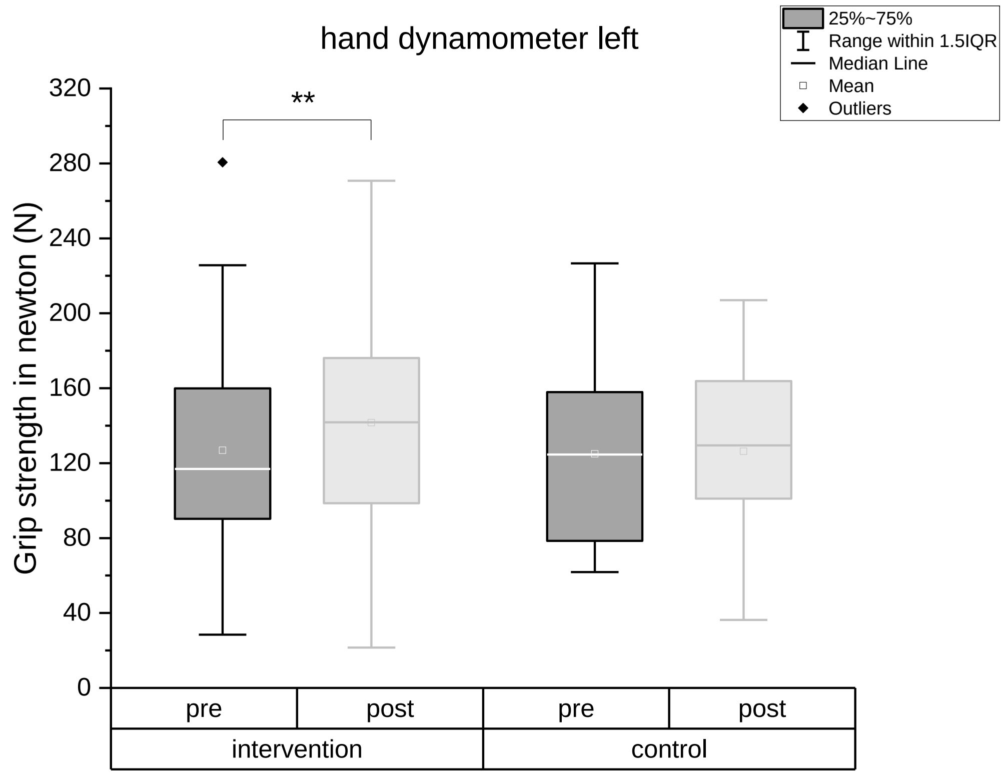

Fig. 9 *Results of the intervention group and control group in the pre-test and post-test at the hand dynamometer left*

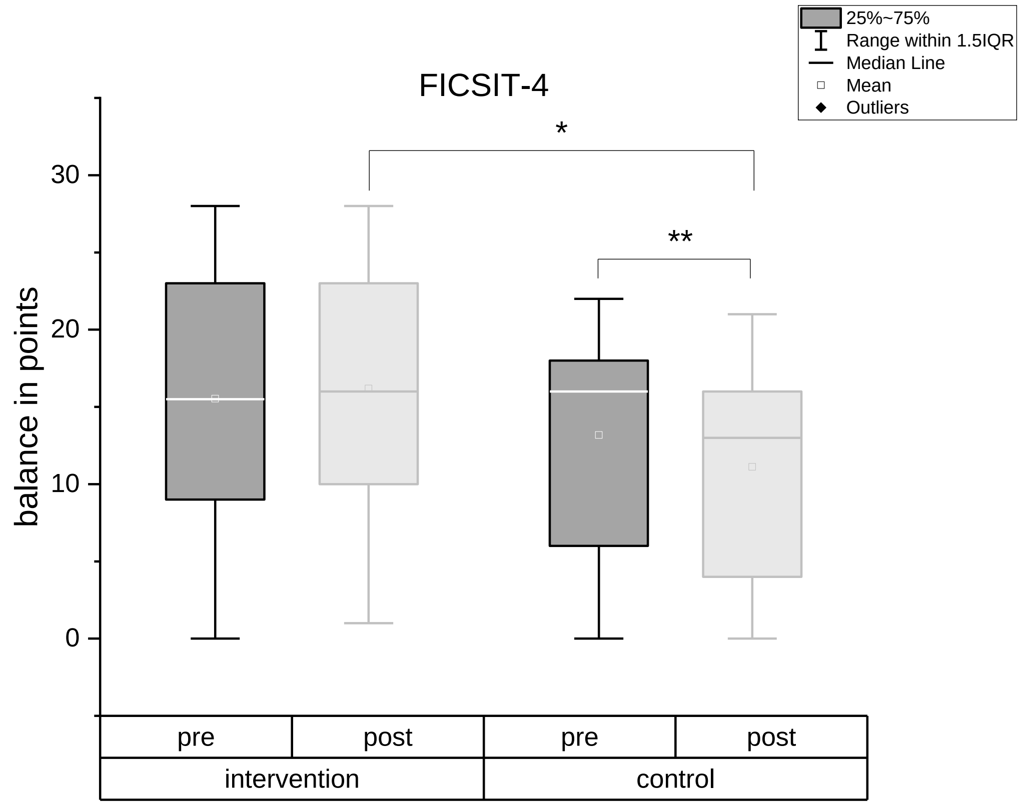

Fig. 10 *Results of the intervention group and control group in the pre- and post-test at Ficsit 4*

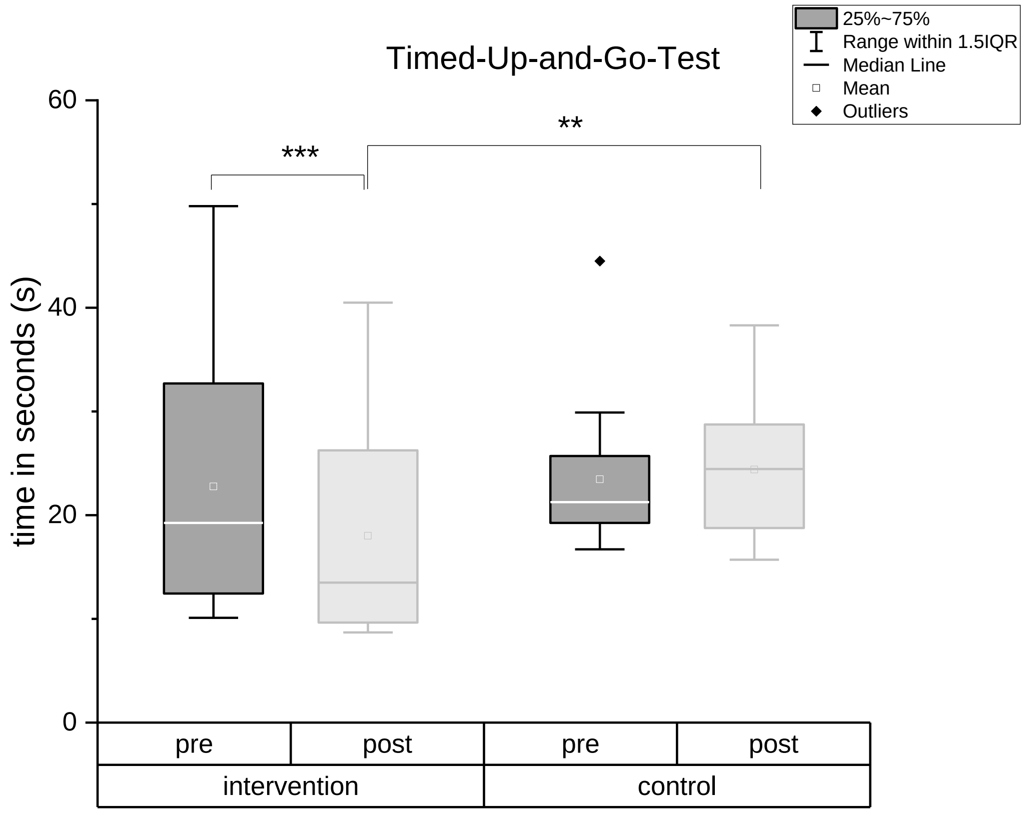

Fig. 11 *Results of the intervention group and control group in the pre- and post-test in the timed-up-and-go test*
