# Supplementary material for: Influence of a multidimensional music-based exercise program on selected cognitive and motor skills in dementia patients—a pilot study
Source: Ger J Exerc Sport Res. 2021 Oct 15;51(4):495–505. doi: 10.1007/s12662-021-00765-z (PMC8519499; doi:10.1007/s12662-021-00765-z)
Supplement: Supplementary file 4 — 4. Sample characteristics [file 12662_2021_765_MOESM4_ESM.pdf]

Influence of a multidimensional music-based exercise program on selected cognitive and motor skills in dementia patients.

German Journal of Exercise and Sport Research

Prinz A.1, Schumacher A.1, Witte K.1
1 Department of Sport Science, Otto-von-Guericke University Magdeburg, Magdeburg, Germany

Corresponding author

Alexander Prinz M.Sc.
Zschokkestr. 32
39104 Magdeburg
0391/67-54862
alexander.prinz@ovgu.de
ORCID: 0000-0003-4416-7913

Sample characteristic

Table 1

Sample characteristics

Baseline characteristics	intervention group (n=32)	control group (n=17)	p-value
age (years, mean ± SD)	83.91 ± 5.73	83.06 ± 6.76	.789
Size (m, mean ± SD)	1.61 ± 0.1	1.60 ± 2.88	.305
Weight (kg, mean ± SD)	70.77 ± 16.35	72.17 ± 12.82	.320
BMI (kg/m², mean ± SD)	27.07 ± *5.71*	28.08 ± 4.68	.433
Sex (%)	m: 15.2 %<br>w: 84.8 %	m: 12.5 %<br>w: 87.5 %	.804
Years in a nursing home (mean ± SD)	1.88 ± 2,8	3.50 ± 2.07	.041
MMSE (score, mean ± SD)	16.66 ± 6.73	18.25 ± 6.90	.380
	Degree of dementia		
no dementia (%)	1 (3.1 %)	1 (5.9 %)	.157
mild dementia (%)	14 (43.8 %)	7 (41.2 %)	.397
moderate dementia (%)	15 (46.9 %)	7 (41.2 %)	.400
severe dementia (%)	2 (6.3 %)	2 (11.8 %)	.261
	Forms of dementia		
Alzheimer's dementia (%)	17 (53.1 %)	8 (47 %)	.408
Vascular dementia (%)	4 (12.5 %)	1 (6 %)	.287
Other type of dementia (%)	11 (34.4 %)	8 (47 %)	.382
Attendance at practice sessions in %	93 %	/	
Education years (mean ± SD)	10.72 ± 3.1	9.53 ± 2.88	.583
